# School Food Policies Related to Soft Drink and Fruit Juice Consumption as a Function of Education Type in Flanders, Belgium

**DOI:** 10.3390/ijerph16152718

**Published:** 2019-07-30

**Authors:** Francisca Marro, Peter Bottenberg, Wolfgang Jacquet, Luc Martens

**Affiliations:** 1 Department of Paediatric Dentistry, PAECOMEDIS Research Cluster, Gent University, 9000 Gent, Belgium; 2 Department of Educational Sciences EDWE-LOCI, Faculty of Psychology and Educational Sciences, Vrije Universiteit Brussel, 1050 Brussels, Belgium; 3 Oral Health Research Group ORHE, Faculty of Medicine and Pharmacy, Vrije Universiteit Brussel, 1050 Brussels, Belgium

**Keywords:** Erosive tooth wear, school food policies, Type of education, socioeconomic school background, soft drinks regulations

## Abstract

Recent data on erosive tooth wear (ETW) in Belgium have associated a vocational/technical type of education with ETW risk. Since the role of schools is essential to the promotion of healthy diets, this study aimed to investigate school food policies (SFP) related to soft drink and fruit juice consumption and to detect differences among schools in Flanders, Belgium (BE-F). An online questionnaire related to the control of acidic beverages and promotion of healthy drinking habits was sent to all Flemish secondary schools. For analysis, schools (*n* = 275) were grouped by type of education (vocational secondary education (VSE) and general secondary education (GSE)), and by socioeconomic status. Multiple factor analyses (MFA) were performed to identify schools with a similar SFP profile. Additionally, descriptive analyses were performed to determine other associations. Overall, 44% of schools in BE-F claimed to have written SFP related to the consumption of soft drinks. SFP expressly prohibiting or limiting acidic beverages were significantly more frequent in GSE schools (*p* < 0.05), where a higher economic status was present. This study shows that a considerable group of schools in BE-F have no or incomplete rules concerning acidic beverage consumption. Such rules differ between types of education, with VSE schools reporting less control regarding the consumption of drinks.

## 1. Introduction

School food policies (SFP) play an important role in determining the food environment of children and adolescents. School environments stimulate health behaviors and influence dietary choices [1] since they determine the access, availability, marketing and advertising of food products that adolescents receive [2]. In 2010, the World Health Organization (WHO) emphasized the importance of the school environment [3], and, in Europe, several countries have adopted strict laws related to food items in schools, including the prohibition of marketing, the presence of vending machines and the sale of food/drinks with added sugar [4].

Sweetened and non-sweetened beverages, such as soft drinks and fruit juices, are food products linked to great availability and heavy marketing at schools. The high intake of these products has not only been associated with non-communicable diseases (cardiovascular diseases, type 2 diabetes) [3] but also with oral health problems, such as tooth decay and erosive tooth wear (ETW) [5]. The latter has received considerable attention in the past decade since the literature suggests that there is an increase in the worldwide prevalence of ETW [6]. The harmful effects of the frequent consumption of acidic beverages on dentition are not broadly known by the population. As such, there is a need to educate and promote healthy drinking habits in order to promote oral health.

ETW is a chemical–mechanical process of tooth wear caused by the presence of non-bacterial acids in the oral cavity [7]. The process involves an accelerated loss of dental hard tissue that, if not prevented, could cause significant oral health problems affecting the durability of the dentition [8]. The frequent consumption of acidic beverages has been associated several times with ETW risk [9,10], and evidence indicates that the consumption of more than one can of drink/day should be considered to be a risk indicator for the presence of ETW [11,12]. Representatives of schools and parents must be aware of the potential erosive effect that acidic beverages have on their children’s dentition in order to restrict access to them in the school environment.

A previous study of ETW in Belgium showed that type of education (vocational/technical) was a predictive variable for ETW risk in adolescents (odds ratio (OR): 1.49; 95% CI: 1.03–2.13). A higher prevalence of ETW was shown in students registered to vocational/technical schools (VSE) where a significantly higher intake of acidic beverages occurred and a lower socioeconomic status (SES) was present [11]. This link between education type, SES, and level of soft drink consumption was similarly shown in Norway by Mulic et al. 2012 [13]. In this study, 18-year old Norwegian adults that had followed vocational education had a higher prevalence of ETW compared to the ones that chose general secondary education. Both publications suggest that the type of education might create an environment that influences the drinking habits of students and, as a consequence, the prevalence of ETW.

Local school regulations in Belgium might explain this finding since they seem to play a role in the final decision of the adolescent to consume more healthy snacks and drinks [14,15]. In Flanders, Belgium (BE-F), schools voluntarily adopt the SFP directions proposed by the European Commission, and currently there is no information concerning the control of soft drinks and fruit juices available at schools.

In 2010, 42% of Flemish adolescents reported drinking soft drinks daily [16]. Belgium has one of the highest per capita consumption of acidic beverages (number four in Europe with >120 L/capita/year) [17], and there is an apparent influence of school regulations on adolescents’ dietary decisions [11,14]. Therefore, the present study aimed to investigate the current SFP related to acidic beverages present in schools and to identify possible SFP differences between two lines of education: vocational/technical (VSE) and general secondary education (GSE).

## 2. Materials and Methods

**Figure 1:**
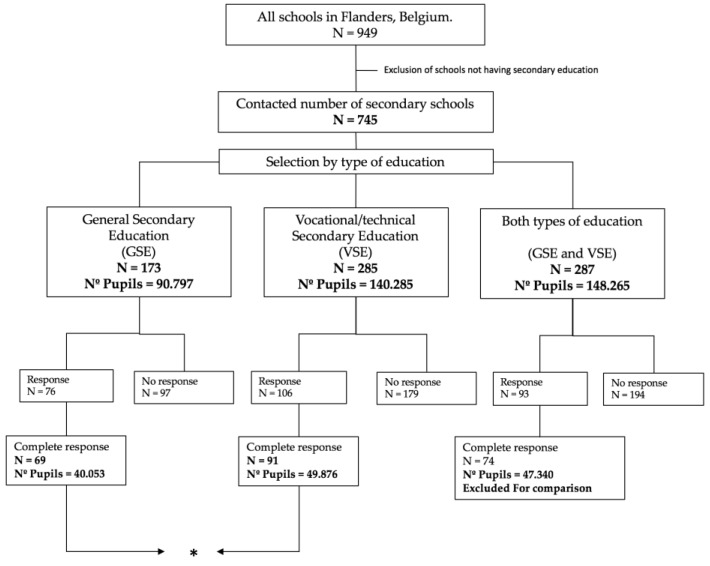
Flow chart of sample recruitment. Only schools that returned a complete response to the questionnaire were used for analysis. (*) indicates which schools were compared, (N) total number of schools and (Nº) number of pupils studying at each type of education.

Between September 2017 and April 2018, a questionnaire aiming to detect SFP related to drinking habits in BE-F was uploaded onto an online platform (Curios) provided by the University of Ghent, and sent via e-mail to the total number of secondary schools listed in the official registers of the Flemish government (*n* = 745; Figure 1). The questionnaire included questions related to the existing written SFP regulations concerning the consumption of soft drinks and fruit juices and the availability of these items at the schools. Additionally, it aimed to detect access to drinkable water at the different institutions and determine the existence of programs that sought to promote healthy drinking habits.

Before the start of the study, three professors from different dental departments and one professor of a methodology department in BE-F (University of Ghent, KU Leuven, and Vrije Universiteit Brussel) performed a content validation of the questionnaire. To increase the response rate, the link containing the survey was sent via e-mail twice (with a reminder sent two months after the first contact with schools).

### 2.1. Sample Selection and Ethical Aspects

From each school, the type of education (GSE versus VSE) and socioeconomic variables, such as the percentage in each school of mothers of students without a secondary education diploma, were recorded. GSE is defined as the type of education that prepares the students for a college/university education, whereas VSE are schools have a less theoretical, but more technical and practical approach compared to GSE. Vocational training aims to prepare students directly for the job market.

The maternal education indicator is one of the four official parameters (maternal education, entitlement to an education allowance, home language, and neighborhood) used by the Belgian educational system to determine the SES of the pupils, and it was chosen among other things in order to allow for comparison with other studies [18,19]. The ethical committee of the Ghent University Hospital indicated that this study did not require the approval of the local ethics committee since it is related to unidentifiable survey data.

### 2.2. Data Management and Statistical Analysis

Data were analyzed using IBM SPSS v. 25.0 (SPSS Inc., Armonk, NY, USA) and R 3.0.3 (The R foundation for statistical computing platform) [20]. A descriptive analysis was performed for every variable. A chi-square test was used to detect associations between the written SFPs and other factors. Additionally, to compare the differences between school types, institutions were grouped according to education type: GSE and VSE. A Mann–Whitney *U* test was used to compare SES differences among the school types. The significance level was set at *p* < 0.05. Furthermore, a multiple factor analysis (MFA) [21] was performed to identify and visualise groups of schools with a similar profile in their answers with respect to written SFP and parental educational level. The representative schools for the clusters were explored with respect to SES and school type. Variables contributing the most to the definition of the different clusters were identified and used for interpretation. All these analyses were performed using the FactoMineR package in R.

Schools containing a mix of education type (GSE and VSE together) were excluded from the comparison analysis shown in Figure 1, and only schools considered exclusively as GSE or VSE were compared in this section. Due to the existence of missing data, a complete case analysis was performed, and schools showing incomplete answers in the questionnaire were excluded from the analysis (15% exclusion).

## 3. Results

### 3.1. Descriptive Statistics

A total of 234 schools, responsible for the education of 137,269 adolescents (total in Flanders 342,458), answered the questionnaire completely and were effectively included in the analysis. From these, 91 were VSE schools and 69 were GSE schools. This sample corresponds to 31.2% of the total number of schools, and is representative of the total population of schools when such variables as SES and school type are considered (Table 1).

### 3.2. SES Differences between Schools

The SES indicator “proportion of mothers without completed secondary education” used in this study differed significantly between school types and indicates that VSE schools have a higher percentage of adolescents whose mothers have no formal education as compared to the mothers of adolescents in GSE schools (Table 1).

### 3.3. School Food Policies Related to Soft Drinks and Fruit Juices

Overall, 44% of the schools in BE-F claimed to have written SFP associated with the consumption of soft drinks in their establishments, quite equally distributed among VSE and GSE schools. SFP concerning fruit juice consumption were found less frequently than those concerning soft drink consumption (Table 1, *p* < 0.01). Two SFP differed significantly (*p* < 0.05) between school type and scored better for GSE schools: Prohibition and limitation of soft drink and fruit juice consumption. (Table 2).

**Figure 2:**
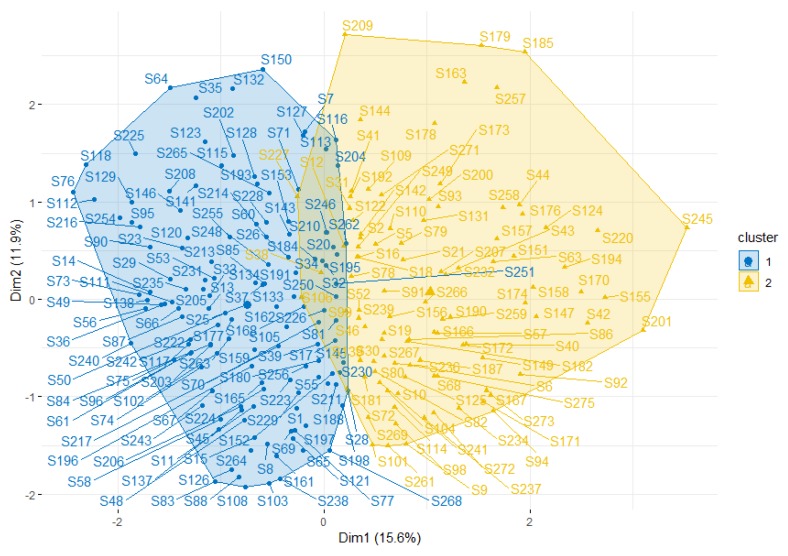
Hierarchical clustering based on multiple factor analysis (MFA) summarising and visualising the data in two-dimensional plots. The blue cluster consists mainly of general secondary education (GSE) schools and the grey cluster mainly represented vocational/technical secondary education (VSE) schools. Row points with a similar profile are closed on the factor map.

An MFA based on the SFP variables showed the existence of two distinguishable groups with a similar profile in their answers (see Figure 2). Variables that contributed the most to each of the two clusters were identified among variables in the data set in order to create a profile of each cluster. The first group mainly represented GSE schools (Figure 2: cluster in blue) and the second group mainly represented VSE schools (Figure 2: cluster in gray). The cluster with predominantly VSE schools was additionally associated with a higher percentage of low-educated mothers while the opposite was observed in the cluster with predominantly GSE schools (a lower rate of low-educated mothers).

### 3.4. Access Times and Availability of Soft Drinks and Fruit Juices

Most of the schools allowed pupils access to soft drinks and/or fruit juices only once a day (65.3%) mainly during the short break time (52.6%), followed by at lunchtime (26.1%). These products were available in 64.9% of institutions, and were offered to pupils mainly through vending machines (VM) (46.6%). Significantly more VSE schools sell soft drinks and fruit juices through VM (*p* < 0.05) (37.6% for GSE and 60.4% for VSE). There were 72 VMs for 40,053 GSE students (1/556.3) and 174 VMs for 49,876 VSE students (1/286.6).

### 3.5. Water Access and Promotion of Healthy Drinking Habits

Most of the schools indicated having access to potable water (92.3%). The promotion of healthy drinking habits was reported by 73.1% of the schools, and it was not statistically significantly different across school types. Promotion was mainly done through the curriculum of the students (30.7%); however, 26% of schools promoted healthy drinking habits using only posters or flyers.

## 4. Discussion

Our previous findings suggested the existence of a possible link between school type, consumption of soft drinks and prevalence of ETW in Belgium [11]. The need to clarify this correlation motivated the present study, which is the first in its kind investigating the SFP related to acidic beverage consumption. The finding that adolescents enrolled in VSE schools consumed significantly higher amounts of soft drinks and presented a significantly higher prevalence of ETW as compared to adolescents enrolled in GSE schools could be partially explained by the school regulations present at the different kinds of education in BE-F. This assumption is of great interest since the role of schools remains essential at the moment to promoting healthy dietary habits during childhood and adolescence [22].

The main outcome of the present study supports the aforementioned results and confirms that SFP associated with acidic beverage consumption differ considerably between school types. GSE schools scored significantly better than VSE schools with regards to prohibiting and limiting soft drinks and fruit juices to their students (Table 1) and, despite the lack of significance among the other variables, overall, GSE schools scored better than VSE schools. The fact that GSE schools prohibit the consumption of acidic beverages might directly influence the adolescents’ dietary choices since in their food environment there is a lack of these products. These SFP differences among school types could explain the consumption patterns previously found in Belgium. Vereecken et al. 2012 [14] indicated that school regulations were associated with the dietary choices of students since schools with restrictive rules against soft drinks were associated with lower consumption of these items at the schools.

Moreover, SES differed significantly among school types, which is similar to what is present in the general population of schools in BE-F [23]. VSE schools had a significantly higher percentage of adolescents whose mothers had a lower level of education as compared to those of GSE schools (*p* < 0.05). The relationship between this SES indicator and school type was further analysed, and in Figure 2 the existence of two main groups answering the questionnaire with a similar pattern and having a significant association with the SES indicator is visualised. Interestingly, one of the clusters was mainly represented by GSE schools with a lower number of students with low-educated mothers, and the other cluster was represented by VSE schools with a higher percentage of students whose mothers have no higher education. Since mothers with lower education demonstrated being more permissive to certain food items according to a previous study performed in Belgium [24], this SES combined with the regulations present in schools could contribute to the final social background that might additionally influence the adolescents’ dietary choices.

The main access to acidic beverages was through vending machines (VM) present in 46.6% of the schools. In this study, GSE schools had significantly less VM at their establishment as compared to VSE schools (37.7% GSE and 60.4% VSE; *p* < 0.05). SFP related to VM sales should be emphasised since, according to the results of this study, they represent the primary source of acidic beverages at Belgian schools. The availability of snacks and soft drinks at schools has been shown to influence the consumption habits of students [1,14]. In Flanders, it is stated that VM at schools should promote the consumption of healthy snacks, including sugar-free drinks. This regulation could reduce obesity, non-communicable diseases, and tooth decay; however, it does not help to control ETW since non-sweetened beverages remain available at the schools. Sugar-free beverages have an erosive potential similar to that of sweetened carbonated soft drinks; therefore, access to both sweetened and non-sweetened beverages should be restricted in future SFP.

The majority of the schools recruited in this study had unwritten regulations related to the consumption of fruit juices (75.6%), whereas for soft drinks it was around 56%. The lack of written rules related to fruit juice consumption might be explained by the assumption that fruit juices are not considered to be an unhealthy item. The current SFP proposed by Union of European Union Soft Drinks Associations (UNESDA) and the WHO aims to decrease the caloric intake in order to reduce obesity among the population. Soft drinks are often linked to obesity problems due to their high sugar content [25] and, therefore, more emphasis is placed on the regulation of this beverage than on that of fruit juices. However, the frequent intake of fruit juices has been considered to be a risk factor for ETW [10,26]. Moreover, in some cases, fruit juices might even have a higher erosive potential than soft drinks [27]. The fact that the majority of schools did not mention fruit juices as part of their regulations indicates a lack of knowledge in this topic that should be considered for the development of future SFP.

The promotion of healthy drinking habits was not significantly different between school types; however, when we analysed the manner of promotion, we found that a main part of the schools promoting healthy drinking habits only used one method, such as posters or flyers. This fact, together with the number of schools that did not promote healthy drinks, indicates that around half of the schools (46.1%) did not properly promote or stimulate healthy drinking habits to their students.

Some limitations should be mentioned in order to allow for a correct interpretation of the results. First of all, due to efficiency reasons, the questionnaire was sent via email, implying the absence of an interviewer, which could reduce the self-response bias associated with surveys. Secondly, despite the fact that all of the schools in Flanders were contacted twice for this study, only 31.2% of them agreed to participate. This could be considered to be a low response rate; however, previous studies performed in Belgium had a similar level of participation [14]. The main refusal reason was that the schools reported being solicited for other investigations during the same academic year. Despite this fact, the sample is proportional to the total population with respect to the two main variables used in this study (SES and school type) and shows the existence of discrepancies regarding SFP present among the school types in Flanders, Belgium.

According to the WHO, the role of schools is essential to promoting healthy dietary habits [22]. The food policies present in schools determine the environment where the young population develops its habits. Since, in BE-F, secondary school is obligatory for everybody, schools have the potential to greatly influence dietary choices throughout adolescence. Therefore, the investigation of the current SFP present in schools remains crucial to develop and implement appropriate SFP in the future that aim to have a positive impact on the dietary habits of the population.

## 5. Conclusions

Less than half of the schools in Flanders were found to have some written regulations concerning soft drink consumption, and almost no control was found to exist regarding fruit juice consumption. The discrepancies found between school types in Flanders indicate that VSE students might be in a disadvantaged position with respect to students belonging to GSE schools because GSE schools tend to prohibit and limit more the consumption of acidic drinks. This outcome might explain a previously shown school effect, which indicated that the consumption of acidic beverages and the presence of ETW was higher in students enrolled at VSE schools. These findings are of great importance for the development of future SFP aimed at establishing similar dietary habits for all types of education.

## Figures and Tables

**Table 1:** Sample Distribution According to School Type and Socioeconomic Status (SES) with Respect to the Total Population.

	All Schools in BE-F *n* = 745		Participating Schools *n* = 234	
	*n* (%)	Proportion of low-educated mothers	*n* (%)	Proportion of low-educated mothers
GSE	173 (23.09%)	13.32%	69 (29.5%)	12.04%
VSE	285 (38.05%)	32.9%	91 (38.9%)	30.07%

Proportion of low-educated mothers differ significantly between GSE and VSE schools *p* < 0.05.

**Table 2:** School Food Policies (SFP) Related to Soft Drink and Fruit Juice Consumption in Flanders, Belgium and Comparison by Education Type.

	Total (*n*:234)	GSE (*n* = 69) General Education	VSE (*n* = 91) Vocational/Technical Education	*p*-Value
The school has written SFP related to the consumption of soft drinks.	44.0% (103)	40.6% (28)	39.7% (36)	0.896
The school has written SFP related to the consumption of fruit juices.	24.4% (57)	27.5% (19)	14.4% (14)	0.06
The school prohibits the consumption of soft drinks and/or fruit juices.	13.2% (31)	18.8% (13)	7.7% (7)	0.035 *
The school limits the consumption of soft drinks and/or fruit juices.	55.1% (129)	65.2% (45)	46.2% (42)	0.017 *
The school allows students to bring their own soft drinks and/ or fruit juices.	85.9% (201)	76.8% (53)	87.9% (80)	0.063
The school allows sale of soft drinks and/or fruit juices to the students	65.0% (152)	65.2% (45)	71.4% (65)	0.401
The school benefits financially from the sale of soft drinks and/or fruit juices at school.	44.0% (103)	46.4% (32)	47.6% (43)	0.912
It is a problem to abandon this sale.	12.8% (30)	14.5% (10)	13.2% (12)	0.812

* *p* < 0.05; Chi-square.
